# Lipid biomarkers in statin users with coronary artery disease annotated by coronary computed tomography angiography

**DOI:** 10.1038/s41598-021-92339-0

**Published:** 2021-06-18

**Authors:** Elena Michelucci, Nicoletta Di Giorgi, Francesco Finamore, Jeff M. Smit, Arthur J. H. A. Scholte, Giovanni Signore, Silvia Rocchiccioli

**Affiliations:** 1 Istituto di Fisiologia Clinica-CNR, via Giuseppe Moruzzi 1, 56124 Pisa, Italy; 2 Department of Cardiology, Leiden University Medical Center, Albinusdreef 2, 2333 ZA Leiden, The Netherlands; 3 Fondazione Pisana per la Scienza Onlus, via Ferruccio Giovannini 13, 56017 San Giuliano Terme, Italy

**Keywords:** Diagnostic markers, Predictive markers, Prognostic markers, Cardiovascular diseases

## Abstract

Molecular markers are suggested to improve the diagnostic and prognostic accuracy in patients with coronary artery disease (CAD) beyond current clinical scores based on age, gender, symptoms and traditional risk factors. In this context, plasma lipids are emerging as predictors of both plaque composition and risk of future events. We aim to identify plasma lipid biomarkers associated to CAD indexes of stenosis severity, plaque lipid content and a comprensive score of CAD extent and its risk. We used a simple high performance liquid chromatography-tandem mass spectrometry method to identify 69 plasma lipids in 132 subjects referred to Coronary Computed Tomography Angiography (CCTA) for suspected CAD, all under statin treatment. Patients were stratified in groups using three different CCTA-based annotations: CTA-risk score, lipid plaque prevalence (LPP) ratio and the coronary artery disease-reporting and data system (CAD-RADS). We identified a common set of lipid biomarkers composed of 7 sphingomyelins and 3 phosphatidylethanolamines, which discriminates between high risk CAD patients and controls regardless of the CAD annotations used (CTA score, LPP ratio, or CAD-RADS). These results highlight the potential of circulating lipids as biomarkers of stenosis severity, non calcified plaque composition and overall plaque risk of events.

## Introduction

Age, gender, symptoms and traditional risk factors are established parameters for pre-test prediction of obstructive coronary artery disease (CAD)^1^. The addition of bio-humoral parameters, such as low-density lipoprotein cholesterol (LDL-C) and high-density lipoprotein cholesterol (HDL-C), triacylglycerol (TG), creatinine, uric acid, gamma-glutamyltransferase (GGT), alanine aminotransferase (ALT), aspartate aminotransferase (AST), white and red blood cells, neutrophils, monocytes, may contribute to improve the discriminatory ability of pre-test scores of obstructive and risky CAD^2,3^ although their additive value is debated^4^.

Reduction in CAD-related mortality observed in patients under optimal medical treatment is largely explained by a better control of cardiovascular risk factors such as smoking cessation, efficient treatment of systemic hypertension, diabetes and dyslipidaemia. In particular, the extensive use of statin therapy to lower LDL-C levels^5^ reduces the risk of developing obstructive CAD and associated adverse events^6^.

Despite the drawbacks and the limits of blood biomarker discovery and their clinical application^7–9^, patient-specific blood phenotype has a great utility in correctly stratifying patients according to CAD related risk as well as in implementing prognostic power of pre-test models^10^. Peptides, hormones and proteins have been intensively investigated in recent years as potential diagnostic or prognostic biomarkers^11,12^. Plasma lipidomics by mass spectrometry is gaining increasing clinical relevance as a source of putative biomarkers of plaque composition and vulnerability. Growing evidences suggest that specific lipids could be predictors of CAD related risk of adverse events^13–17^.

Some reports support a close association between dysregulation of lipids and atherosclerotic lipid burden^14^. However, no study has reported so far the cross-association of dysregulated lipids with an integrated assessment of coronary atherosclerosis in stable CAD patients on statin treatment. Thus, role of circulating lipids as diagnostic biomarkers of coronary atherosclerosis still remains undisclosed.

Non-invasive coronary imaging (anatomical and/or functional) is the first line test for establishing CAD diagnosis^5^ and planning an optimal patient management. The ability to re-stratify patients prior to non-invasive coronary imaging according to risk of significant stenosis and prevalence of potentially vulnerable plaques, would confer important benefits. More aggressive medical therapy would be recommended for those classified at high risk, while a more conservative, lifestyle-based approach would be applied to those subjects classified at low risk, ultimately reducing pharmacotherapy-related costs. Additionally, coronary imaging could be recommended only to patients likely to have coronary atherosclerosis thus limiting radiation exposure.

The aim of this study is to investigate the association between lipid plasma concentration and several Coronary Computed Tomography Angiography (CCTA)-derived indexes of CAD in a cohort of stable patients on standard of care statin treatment. The method used to identify and quantify the lipid biomarkers is a quick and simple selected reaction monitoring (SRM)-based high performance liquid chromatography-tandem mass spectrometry (HPLC-MS/MS) method, an approach which is immediately translatable to clinical practice in view of its high scalability and low cost for analysis. Patient annotation tooks advantage of a recently proposed comprehensive atherosclerotic risk score based on non-invasive CCTA coronary imaging (CTA score)^18^ that integrates plaque extent, plaque location, stenosis and composition, providing high diagnostic yield and prognostic accuracy in patient stratification^19^. We used this score to classify patients into 4 groups, from No CAD (CTA score = 0, normal coronary arteries) to highest severity Class 3 (CTA score > 20).

For comparison, in order to verify the consistency of the association with plasma lipids, we also classified patients according to two other common morphologic indexes of CCTA-assessed CAD: the index of presence of coronary plaques with a prevalent lipid component obtained by a ratio between mixed and non-calcified plaques/total plaques (lipid plaque prevalence ratio, LPP ratio)^14^, and standard CAD-RADS (coronary artery disease-reporting and data system) index^20^ by maximal diameter stenosis.

**Figure 1:**
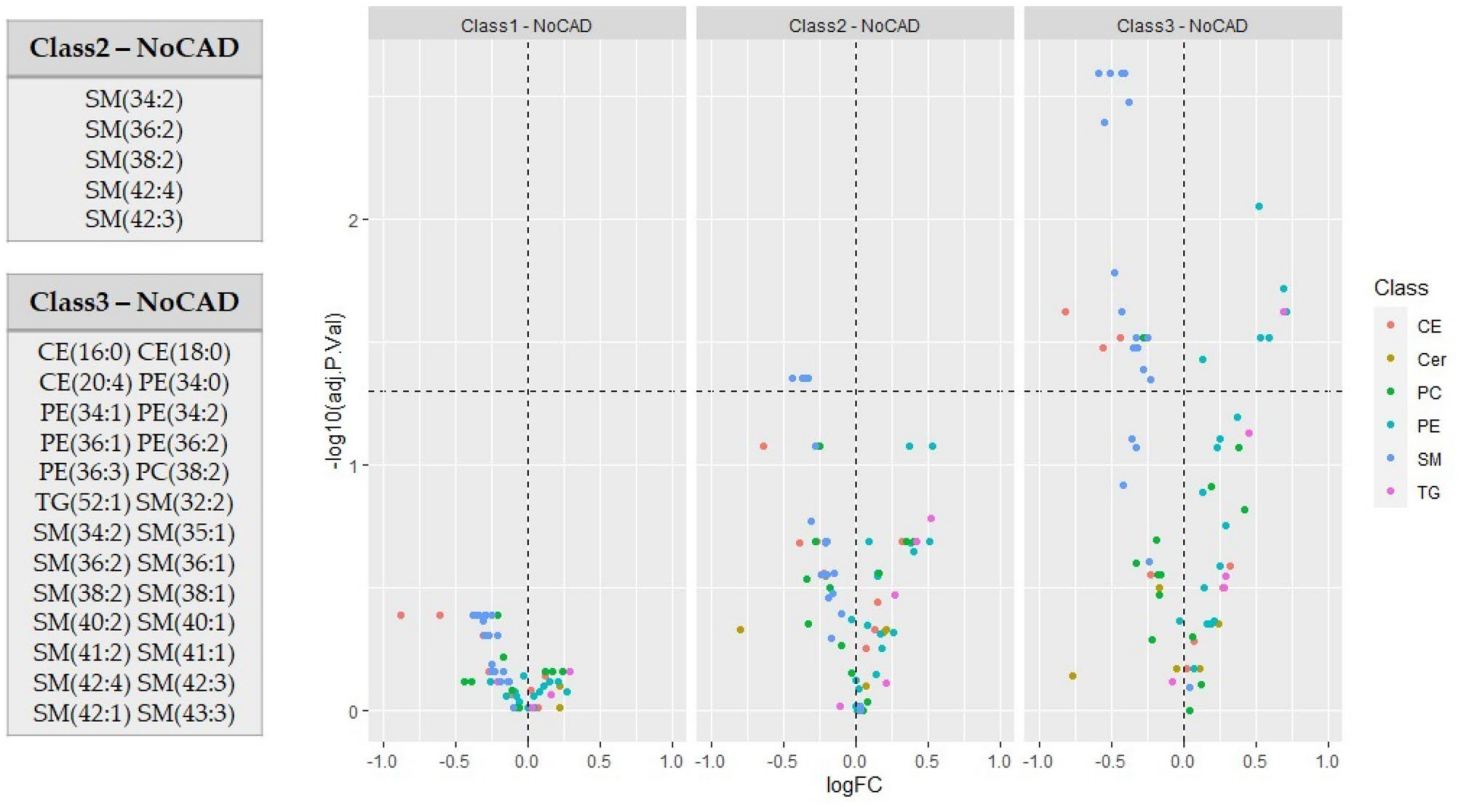
Lipid profile analysis based on CTA score annotations. Volcano plots showing the differentially expressed lipids (adj. p-value threshold = 0.05) among CTA score annotation Classes relative to No CAD subjects. Lipids are colored according to lipid class (CE, Cer, PC, PE, SM, TG). The panels on the left report in details lipids that are significantly dysregulated in comparison Class 2 vs No CAD and Class 3 vs No CAD.

**Figure 2:**
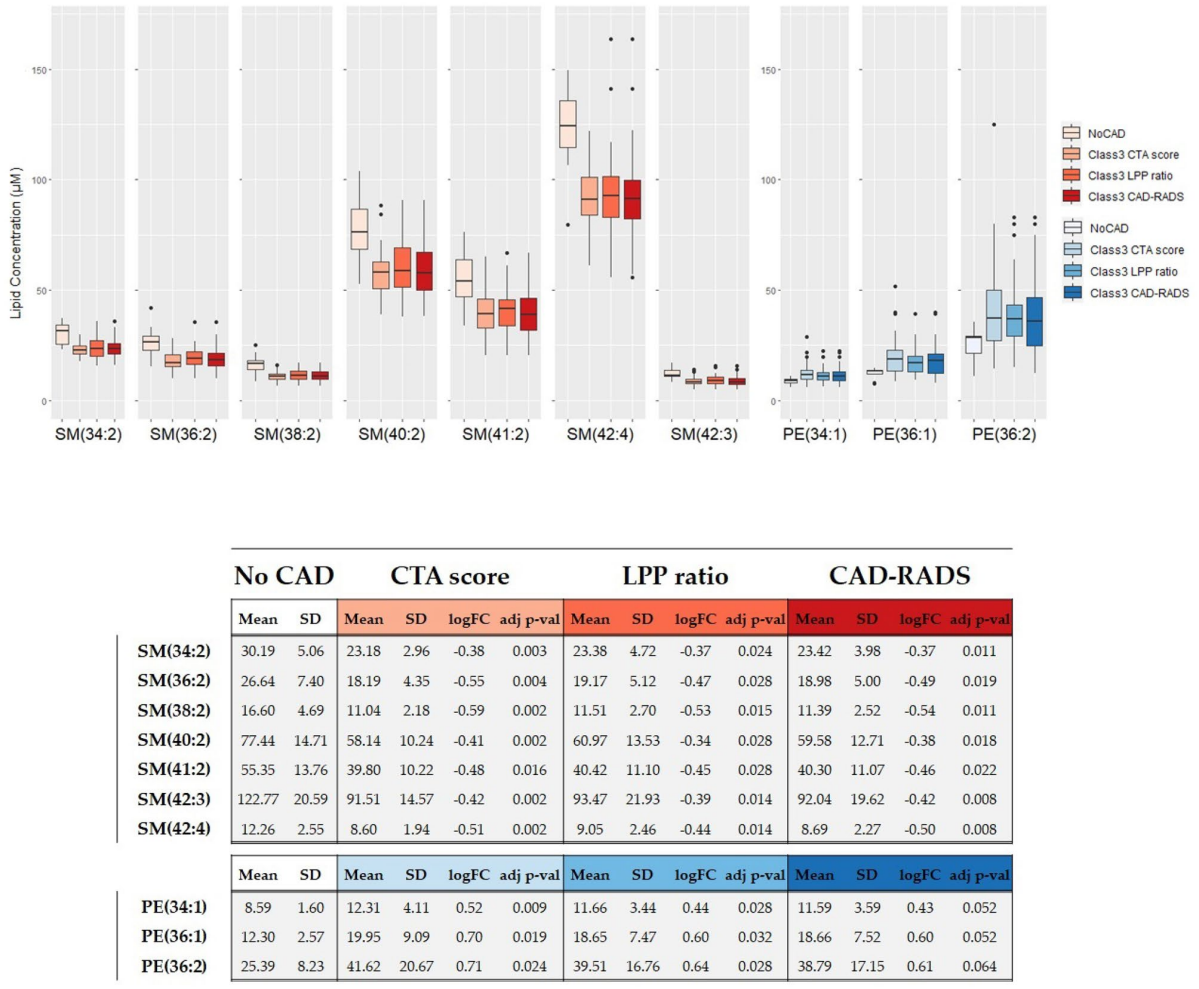
Box plots reporting plasmatic concentration (μM) distribution for the panel of lipid biomarkers consisting of 7 down-regulated SMs and 3 up-regulated PEs in groups of patients with a more severe and vulnerable form of CAD (Class 3 defined by 3 different annotations: CTA score, LPP ratio and CAD-RADS) with respect to control subjects (No CAD patients). The table below reports respective means, standard deviations (SD), degrees of variation (evaluated as log$_2$ transformed fold change) and statistical significance (adjusted p-value) for the 7 SMs and 3 PEs.

**Figure 3:**
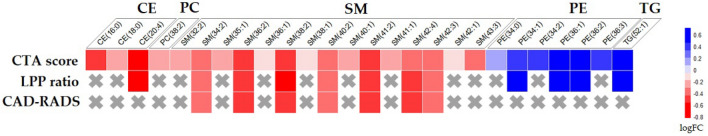
Scheme representative of differentially expressed lipid molecules obtained in extreme comparisons (i.e. Class 3 vs No CAD for CTA score, CAD RADS and LPP ratio). The number of significative dysregulated lipids decrease from CTA score (26 lipids) to LPP ratio annotation (12 lipids), and finally to CAD-RADS (7 lipids), gradually selecting a narrower panel of dysregulated lipids. Although all annotations show concordant results, CTA score seems to be the best classification to obtain the most informative result from circulatory lipids. The color intensity represents logFC values obtained in the extreme contrast (Class 3 vs No CAD) for the 3 annotations.

## Results

We quantified 69 circulating lipids (Supplementary Table S1 in Supplementary Information) significantly related with CAD characterization and prognosis^13–17^ by using a SRM HPLC-MS/MS approach encompassing six lipid classes and subclasses (triacylglycerol [TG], phosphatidylcholine [PC], phosphatidylethanolamine [PE], ceramide [Cer], sphingomyelin [SM], cholesterol ester [CE]).

Data analysis of these lipids was performed with the aim of identifying lipids associated to CAD, assessed by CTA score, CAD-RADS or LPP ratio classifications. In all annotations, patients with normal coronary arteries at CCTA analysis (N = 10) were grouped separately and used as control (No CAD).

The analysis of the groups defined by CTA score annotation evidences twenty-six significantly dysregulated lipids in Class 3 compared to No CAD. 15 SMs, 3 CEs and PC(38:2) are down-regulated, while 6 PEs and TG(52:1) are up-regulated. Some of dysregulated SMs are significantly down-regulated also in Class 2 compared to No CAD. No differences are evidenced comparing Class 1 with No CAD subjects (Fig. 1). For CTA score a non-categorized analysis was also performed showing consistent results (data not shown).

According to LPP ratio based analysis no differences are observed between Class 0 and No CAD as expected by the literature^14^, while twelve lipids are significantly dysregulated in Class 3 compared to No CAD. 7 SMs and CE(20:4) are down-regulated, while 3 PEs and TG(52:1) are up-regulated. Among those, PE(36:1) and 6 SMs are also dysregulated in Class 2 and Class 1 respectively compared to No CAD (Supplementary Fig. S1, Supplementary Information).

Finally, according to CAD-RADS annotation, 7 SMs are down-regulated in Class 3 compared to No CAD. Four of these are significantly down-regulated also in Class 2 compared to No CAD. This last comparison evidences five additional dysregulated lipids: CE(20:4), PE(34:1), PE(36:1), PE(36:2) and TG(52:1) (Supplementary Fig. S2, Supplementary Information).

Interestingly, a common panel of lipids seems to differentiate statin treated patients with a more severe and vulnerable form of CAD (Class 2 and Class 3 for the three annotations) compared with statin treated control subjects (No CAD patients): 7 down-regulated SMs [SM(34:2), SM(36:2), SM(38:2), SM(40:2), SM(41:2), SM(42:4), SM(42:3)] and 3 up-regulated PEs [PE(34:1),PE (36:1),PE (36:2)] (Fig. 2). In addition, CE(20:4) and TG(52:1) are respectively down- and up-regulated in at least one comparison performed in all the three different analyses.

## Discussion

In this paper we assessed the association between CAD indexes of stenosis severity, composition and risk and a set of circulatory lipid biomarkers in stable CAD patients on optimal medical therapy. The vast majority of CAD patients is on standard of care statin treatment to maintain LDL-C levels within recommended limits, but a detailed lipidomics analysis in stable CAD patients under this treatment and using different CAD classification methods has never been reported.

Among the scores proposed to classify CAD patients, CTA score has been recently demonstrated to be the most accurate in combining disease severity and risk^19^. It is an integrated index of CAD extent that encompasses number of plaques, their location in coronary tree, composition (non-calcified vs mixed or calcified) and stenosis severity, with a documented high prognostic value for clinical outcomes in stable CAD patients^19^. Thus, the main annotation used in this work is the comprehensive CTA score, although two other CCTA-based annotations related to CAD (LPP ratio and CAD-RADS) were considered for comparison.

CAD-RADS is the clinical recognized standard classification based on maximal stenosis degree for CAD diagnosis. It is the only used to support clinical decision on interventional treatment of stable CAD according to current guidelines. LPP is another morphological index of atheroscerotic lipid burden, which is a feature of plaque vulnerability^14^.

We highlight that the largest and more complete pattern of circulatory lipid biomarkers associated with stable CAD patients is obtained using CTA score classification (26 lipids). Comparison of Class 3 with control group using any of the other two CCTA annotations (LPP ratio and CAD-RADS) evidences a lower number of significatively dysregulated lipids, that decreases from LPP ratio (12 lipids) to CAD-RADS annotation (7 lipids) (Fig. 3).

Our results are in line with current literature. Since 2006, when mass spectrometry based lipidomics was firstly used to evaluate levels of single SM species, circulatory SM were found associated with long-term mortality^21^. However, to date there are no reports suggesting their positive or negative association with CAD stenosis, extension and composition. It is known that inflammatory conditions, such as atherosclerosis, can increase sphingomyelinase (SMase) activity, breaking down sphingomyelins and reducing their level. Plasma SM levels could be a marker for atherogenic remnant lipoprotein accumulation and may predict lipoprotein susceptibility to arterial wall^22^. Additionally, SM species are reported to be the second most abundant phospholipid component and the major sphingolipid in HDL-C particles^23^, that have a crucial role in atheroprotection. Dysfunctional HDL-C exhibits 25% less lipids per milligrams of protein, reflecting lower contents of SM and PC, and a substitution of 50% of CE for TG^24^. These lipid changes can alter antiatherogenic HDL-C assets, reducing their cholesterol efflux capacity and hindering reverse cholesterol transport.

Finally, an important positive association of circulatory PEs with atherosclerosis extension and progression was demonstrated in the article of Dang et al. in an extensive analysis of plasma lipids in ApoE−/− mice^25^. The majority of the studies proposed in the literature demonstrate the association between circulating lipids (from TG, CE, PC, PE, Cer classes) and long term cardiovascular outcomes^16,17^. Lipidomics derived test scores, such as the SMART risk score^26^ and CERT2^27^, are widely recognized for their prognostic value for any type of cardiovascular event. In few clinical trials, circulatory lipidomics is associated with coronary plaque severity and composition evaluated by invasive and/or non-invasive imaging. Significant associations with non-calcified coronary artery plaque burden assessed by CCTA have been reported^14^, demonstrating the role of circulatory lipids (mainly PEs and CEs) on total atherosclerosis burden of non calcified plaques.

In this work we evaluated the association of stenosis severity and non calcified plaque composition with circulatory lipids. These represent potential residual atherogenic molecules despite the recommended statin treatment^28^, which should promote plaque calcification^29^, leading to stabilization and reduction of lipid-rich plaques^14^.

The results of this study in statin users demonstrate that stenosis severity and atherosclerotic lipid burden are significantly associated with specific lipid biomarkers among the classes of SM and PE. This finding is consistent with a role of lipid biomarkers in atherogenesis and CAD development, prompting their exploitation as predictors of disease state.

The limited number of patients examined in this study implies that more extensive validation on a larger cohort of patients will be required to fully corroborate our findings. However, we underline that patients clinical characteristics were similar between CAD and control groups, the sole differences being age and gender. A further parameter that could require more extensive validation is represented by the effect of statins, whose different nature and administered dose could exert a not completely predictable effect on circulating lipids.

In conclusion, we identified a common set of lipid biomarkers composed of 7 SMs and 3 PEs , which discriminates between high risk CAD patients and controls regardless of the CAD annotations used (CTA score, LPP ratio, or CAD-RADS). We also observed that, as expected based on the number and nature of the underlying parameters, classification based on CTA score is cross-associated with a greater number of dysregulated lipids in blood.

This set of lipid biomarkers opens the way to the possibility to identify patients with a more extensive lipid plaque burden and severe coronary stenosis by a simple targeted lipid analysis from plasma. In perspective, this could improve patient management throughout more aggressive primary prevention strategies where necessary, possibly leading to a quick and easy identification of “vulnerable” patients at risk of future events.

## Methods

### Study design, patient population and ethical approval

Plasma samples of 132 statin users at intermediate/high risk of obstructive CAD according to ESC guidelines on chronic coronary syndrome^5^ participating to the clinical study of H2020-SMARTool project, were collected together with clinical, demographic characteristics and clinical biochemistry. All patients referred to Radiology Departments for CCTA. No substantial differences in statin type (Simvastatin, Atorvastatin, Rosuvastatin) were present, but there was a predominant use of Atorvastatin (43.2% of population). Statins were administered 6.3 ± 1.4 years before their enrollment with a medium-high dosage (Atorvastatin $\ge$ 10 mg/die, Rosuvastatin $\ge$ 5 mg/die, Simvastatin $\ge$ 20 mg/die) for the 84% of the population.

The study was conducted according to the Declaration of Helsinki and its later amendments. The Ethical Commitee of Area Vasta Nord Ovest (Italy) approved the Multicentric European Study under Coordination of Fondazione Toscana Gabriele Monasterio Hospital in Pisa (7 countries involved, Clinicaltrial.gov Identifiers NCT04448691). Written informed consent was obtained from all individual participants included in the study.

### Coronary computed tomography angiography patient classification

Subjects underwent CCTA scan using 64-slice scanners or higher, according to the predefined standard operating procedure of SMARTool Project Clinical Study, in order to ensure optimal image quality. All CCTA images were analysed blinded to clinical data by a separate Core Laboratory (Leiden University Medical Center) and coronary arteries were assessed according to the modified 17-segment American Heart Association classification^30^.

The CTA score was calculated according to what reported in the literature^18^ and it is the per-patient sum of the scores of the 17-segments analysed, combining plaque composition (calcified, non-calcified, and mixed plaque) with stenosis severity and location in each segment applying a weight factor. Besides subjects with normal coronary arteries and CTA score = 0, patients were classified into 3 groups, Class 1 (score < 5), Class 2 (score 5-20) and Class 3 (score > 20), using CTA score thresholds as reported in the literature^19^.

Mean plaque composition was qualitatively assessed as non-calcified, mixed or calcified by using both fixed (− 30 to 130, 131 to 350 and $\ge$ 351 HU density value, respectively) and adaptive, luminal contrast density-corrected thresholds, as reported^31^. Lipid plaque prevalence (LPP), was defined by assessing the prevalence of mixed and non-calcified plaques, calculated as the ratio to the total number of plaques in each patient^14^. The ratio (non-calcified + mixed plaques)/total number of plaques was used to group patients into low (Class1), medium (Class2) and high (Class3) prevalence of soft plaques according to the tertiles of the values distribution in our population and named by us lipid plaque prevalence ratio (LPP ratio). In this last annotation we defined a Class 0 as the patients without mixed or non-calcified plaques in the coronary tree.

The CAD-RADS classes have been used to group subjects into four main categories of stenosis severity, No CAD (0% stenosis), Class 1 (< 30% stenosis), Class 2 (30–50% stenosis) and Class 3 (> 50% stenosis), corresponding to normal coronary arteries, minimal CAD, non obstructive CAD and obstructive CAD respectively^32^.

Patient group characteristics are reported in Tables S3, S4 and S5 (Supplementary Information).

### Chemicals and materials

Formic acid (56302) and ammonium formate (14266), both eluent additives for high performance liquid chromatography-mass spectrometry (HPLC-MS), were purchased from Fluka Analytical (Sigma-Aldrich, St. Louis, MO, USA). Methanol, propan-2-ol (both ultra-purity solvents) and chloroform (super purity solvent) were purchased from Romil (Waterbeach, Cambridge GB-CB25 9QT). NaCl (S3014) and DMSO (276855) were purchased from Sigma-Aldrich (St. Louis, MO, USA). Milli-Q deionized water was filtered on Millipak filter (0.22 m, MPGL040001) and purified on a LC-Pak cartridge (C18, LCPAK0001) (all Millipore, Bedford, MA 01730, USA).

Lipid calibration standards were the following: CE(17:0) (64-1700) purchased from Larodan (Solna, Sweden); TG(17:0/17:0/17:0) (T2151) and SM(d18:1/16:0) (91553) purchased by Sigma Aldrich (St. Louis, USA); Cer(d18:1/17:0) (860517P), 1,2-PC(17:0/17:0) (850360), 1,2-PE(15:0/15:0) (850704) all from Avanti Polar Lipids (Alabaster, USA). N,N-dimethylsphingosine (d18:1) (DMS) (860496O), purchased from Avanti Polar Lipids (Alabaster, USA), was selected as internal standar (ISTD).

### Lipid analysis by direct infusion and calibration curves

Six stock solutions of lipid calibration standards were prepared at concentration of 1.5 mM by dissolving accurately weighted lipid powders in the following solvents: CHCl$_3$/MeOH 70/30 for Cer(d18:1/17:0) and 1,2-PE(15:0/15:0); CHCl$_3$ for CE(17:0), TG(17:0/17:0/17:0), SM(d18:1/16:0) and 1,2-PC(17:0/17:0). The ISTD stock solution was prepared by diluting a known volume of *N*,*N*-dimethylsphingosine in DMSO to a final concentration of 15.3 mM. The seven stock solutions were stored at − 20 °C in the dark until use. Preparation, storage and subsequent manipulation of these solutions were performed using glass/stainless steel syringes and glass vials.

The seven stock solutions were first diluted in MeOH with 0.1% HCOOH and 1 mM ammonium formate to the final concentration of 1.5 μM and then their full scan and collision induced dissociation (CID) spectra were recorded by direct infusion at 10 μl/min flow rate (details in Supplementary Information, see “MS analyses: direct infusion and HPLC-MS/MS conditions” paragraph and Supplementary Table S1).

First, ISTD stock solution was diluted with MeOH/CHCl$_3$ 1/2 to a concentration of 1.67 μM . Then, sixteen calibration levels, ranging from 222.2 to 0.007 µM, were prepared for each calibration STD according to the following procedure. A volume of 29.6 µl of calibration STD stock solution was added to 160.4 µl of MeOH/CHCl$_3$ 1/2 (final volume 190 µl). This solution was split in two aliquots of 95 µl each. Five µl of 1.67 µM DMS were added to the first aliquot, thus creating the first calibration level (222.2 µM calibration STD, 0.084 µM ISTD), while the second one was two-fold diluted with MeOH/CHCl$_3$ 1/2. The latter solution was in turn split in two aliquots of 95 µl each and sequential two-fold dilutions were carried out till the 16th calibration level (0.007 µM calibration STD, 0.084 µM ISTD). Only in the case of calibration STDs 1,2-PC(17:0/17:0), TG(17:0/17:0/17:0), CE(17:0) and SM(d18:1/16:0), the sixteen calibration levels were further five-fold diluted with MeOH/CHCl$_3$ 1/2 (first calibration level 44.4 µM, last calibration level 0.0014 µM, all levels with an ISTD concentration of 0.0168 µM). Finally, a specific range of calibrators was selected for each STD in order to build the corresponding calibration curve: 0.007–0.87 µM for Cer(d18:1/17:0); 0.027–13.9 µM for 1,2-PE(15:0/15:0); 0.011–22.2 µM for 1,2-PC(17:0/17:0); 0.011–11.1 µM for TG(17:0/17:0/17:0); 0.043–44.4 µM for CE(17:0); 0.005–5.56 µM for SM(d18:1/16:0).

### Plasma sample collection and preparation

Patient plasma samples, stored at − 80 °C, were thawed at room temperature and immediately subjected to lipid extraction and analysis. Total lipid extraction from an aliquot of plasma was performed according to Folch procedure^33^: 50 µl of sample were put in a 1.5 ml microcentrifuge tube and were diluted with 100 µl of 150 mM NaCl aqueous solution and 600 µl of 0.0625 µM DMS in MeOH/CHCl$_3$ 1/2. The biphasic solution thus formed was incubated at 25 °C for 30 min at 1000 rpm in a Thermomixer Compact (Eppendorf, Hamburg, Germany) and then centrifuged at 13,000 rpm for 10 min at 10 ºC in a Microcentrifuge Heraeus Biofuge Fresco (Thermo Scientific, MA, USA). From each sample two aliquots of the lower phase were transferred into glass HPLC vials for the subsequent HPLC-MS/MS analysis: the first aliquot was analyzed as such while the second one was five-fold diluted with MeOH/CHCl$_3$ 1/2. ISTD concentration was 0.0625 µM in 600 µl of MeOH/CHCl$_3$ 1/2 used in the Folch procedure and consequently, after lipid extraction, 0.084 µM in 450 µl of organic bottom layer.

### HPLC-MS/MS analysis

STD calibration curve and plasma sample analyses were performed by liquid chromatography-electrospray ionization-tandem mass spectrometry (details in Supplementary Information, “MS analyses: direct infusion and HPLC-MS/MS conditions” paragraph). Different volumes of calibration STDs/plasma samples were injected in order to avoid mass spectrometer saturation due to CE, TG, SM and PC species: 0.5 µl for STDs 1,2-PE(15:0/15:0) and Cer(d18:1/17:0); 0.1 µl for STDs CE(17:0), TG(17:0/17:0/17:0), SM(d18:1/16:0) and 1,2-PC(17:0/17:0); 0.5 µl for plasma samples as such and 0.1 µl for plasma samples diluted five-folds, respectively for the quantitation of PE/Cer and CE/TG/SM/PC species.

To prevent carry-over two needle wash solutions (MeOH/i-PrOH 50/50 and MeOH/CHCl$_3$ 1/2) were used and blanks were placed at the end of each calibration curve (after the higher calibration level) and after each sample. In this way we only observed a minimal carry-over of TGs, negligible for all practical purposes (< 0.1% in all cases).

Injections were repeated in triplicate for each lipid standard. Calibration curves and their R$^2$ are reported in Supplementary Table S2. Lipid absolute concentrations were calculated considering their area ratio (lipid peak area/ISTD peak area) and interpolating it within the calibration curve of the corresponding external standard. MultiQuant 2.1 software (SCIEX, Canada) was used for lipid quantification (Supplementary Table S6).

### Statistical analysis

The clinical characteristics and clinical biochemical parameters of patients classified by CCTA annotation (CTA score, LPP ratio and CAD-RADS) are listed in Supplementary Information Tables S3–S5.

Continuous variables were compared between groups using one-way ANOVA test, followed by a two-tailed Student T-test with Bonferroni correction in order to compare each group of subjects with respect to the control group (No CAD Class) only for the variable resulted significantly different (ANOVA p-value < 0.05). Otherwise, categorical variables were compared between groups using Chi-square test. Data were analyzed using SPSS Statistics software (IBM, version 26).

Quantitative data of 69 lipids were used to investigate differences in groups of CAD severity (by CTA score and CAD-RADS) and plaque composition (LPP ratio). Kolmogorov-Smirnov test was used to assess how close the data are to a normal distribution. As the data were not normally distributed (p-values < 0.05), two-tailed Mann–Whitney U-test was performed in order to consider the significant differences in lipid levels between the different annotation groups with respect to the control group (No CAD patients). P-values were corrected using the Benjamini–Hochberg procedure in order to minimize any type I error and thus the occurrence of false positives. Lipids differences were considered statistically significant with a corrected p-value lower than 0.05 for all analysis. Lipidomics experimental data were analyzed using R software (version 3.6.3).

## Supplementary Information

Supplementary Information.
