# Supplementary material for: Lipid biomarkers in statin users with coronary artery disease annotated by coronary computed tomography angiography
Source: Sci Rep. 2021 Jun 18;11:12899. doi: 10.1038/s41598-021-92339-0 (PMC8213699; doi:10.1038/s41598-021-92339-0)

Supplementary Information

Title: Lipid biomarkers in statin users with coronary artery disease annotated by Coronary Computed Tomography Angiography

Authors: Elena Michelucci[1,*,+], Nicoletta Di Giorgi[1,+], Francesco Finamore[1], Jeff M. Smit[2], Arthur J. H. A. Scholte[2], Giovanni Signore[3,$], Silvia Rocchiccioli[1,$,*]

[1]Istituto di Fisiologia Clinica-CNR, via Giuseppe Moruzzi 1, 56124, Pisa, Italy

[2]Department of Cardiology, Leiden University Medical Center, Albinusdreef 2, 2333 ZA Leiden, The Netherlands

[3]Fondazione Pisana per la Scienza Onlus, via Ferruccio Giovannini 13, 56017, San Giuliano Terme, Italy

[*]Correspondence: silvia.rocchiccioli@ifc.cnr.it and emichelucci@ifc.cnr.it; Tel.: +39 050 3153093; Fax: + 39 050 3152626

[+]These two authors contribute equally to this work

[$]These two authors are co-last authors

Table of contents

MS analyses: direct infusion and HPLC-MS/MS conditions 3

Table S1. Table used for the Scheduled MRM analysis of the 7 STDs and the Scheduled SRM analysis of 69 lipid species 4

Table S2. Equations and R^2 obtained for the calibration curves of lipid classes 9

Table S3. CTA score: classification and patient characteristics 10

Table S4. LPP ratio: classification and patient characteristics 12

Table S5. CAD-RADS: classification and patient characteristics 13

Table S6. Means and standard deviations for 69 quantified lipid species among groups of patients defined by CTA score annotations. 14

Figure S1. Lipid profile analysis based on LPP ratio annotation. 18

Figure S2. Lipid profile analysis based on CAD-RADS annotation 19

MS analyses: direct infusion and HPLC-MS/MS conditions

Direct infusions of seven STD solutions were performed in a 5500 QTrap mass spectrometer (SCIEX, Concord, Ontario, Canada) equipped with a Turbo V ESI (electrospray ionization) source. Working conditions were set as follows: positive polarity, mass range 100-1000 *m/z* (both for full scan and CID (collision induced dissociation) spectra), curtain gas 20 psi, ion spray voltage 5 kV, probe temperature 200 °C, ion source gas 1 and gas 2 10 psi, declustering potential 100 V, entrance potential 10 V. CID experiments were performed by using N_2 as collisional gas, the collision cell exit potential was 19 V, while the optimized collision energies (CEns) for each STD and the relative product ions chosen are reported in Table S1. Analyst Software 1.6.3 (SCIEX) was used for acquisition.

HPLC-MS/MS (high performance liquid chromatography-tandem mass spectrometry) analyses of external standard (STD) calibration curves and plasma samples were performed using a Nexera X2 HPLC system (Shimadzu, Kyoto, Japan) combined with a QTrap 5500 mass spectrometer (SCIEX, Concord, Ontario, Canada) equipped with a Turbo V™ ESI source. Vials were put in a refrigerated autosampler at 5 °C. Two needle wash solutions were used: MeOH/i-PrOH 50/50 and MeOH/$CHCl_3$ 1/2. Gradient elution at a flow rate of 0.2 ml/min was performed on a Kinetex column packed with C8 phase (100 × 2.1 mm, 1.7 µm, 100 Å) equipped with a guard column packed with C8 phase (3 mm ID), both Phenomenex (Torrance, CA, USA). Mobile phase A was MeOH/H_2O/i-PrOH 50/45/5 with 0.1% HCOOH and 1 mM ammonium formate; phase B was MeOH/i-PrOH 50/50 with 0.1% HCOOH and 1 mM ammonium formate. The gradient elution program was: 0 min, 65% B; 0.5 min, 65% B; 10 min, 100% B; 15 min, 100% B; 15.1 min, 65% B; 20 min, 65% B. The column temperature was 45 °C. The mass spectrometer was set in positive ion mode and the operation conditions were the following: curtain gas 30 psi, ion spray voltage 5 kV, probe temperature 200 °C, ion source gas 1 and gas 2 30 psi, declustering potential 100 V, entrance potential 10 V, collision cell exit potential 19 V, collisional gas N_2. The optimized CEns for each STD/lipid species, and the relative product ions chosen, are reported in Table S1. Selected reaction monitoring data acquisition were accomplished using the Scheduled MRM (multiple reaction monitoring) Algorithm in Analyst Software 1.6.3 for 69 lipid species (SCIEX, Concord, Ontario, Canada) with a fixed cycle time of 1.5 sec.

Table S1. Table used for the Scheduled MRM analysis of the 7 STDs and the Scheduled SRM analysis of 69 lipid species (fixed cycle time 1.5 sec).
TG = triacylglycerol, PC = phosphatidylcholine, PE = phosphatidylethanolamine, CE = cholesteryl ester, SM = sphingomyelin, Cer = ceramide, ISTD DMS = internal standard N,N-dimethylsphingosine (d18:1). The *m/z* values are calculated using the monoisotopic molecular weights. Q1 = first quadrupole of the mass spectrometer for precursor ion selection. Q3 = third quadrupole of the mass spectrometer for product ion selection**.** For the STDs are reported the optimized CEns in order to generate their quantifier and qualifier transitions. The transitions selected for the 69 lipid species were based on the quantifier ion of the corresponding external standard. R = alkyl chain of the fatty acid (of the amide group in the case of SM and Cer STDs). Window = defines the window of time (centered around the retention time) for which the transition is monitored.

[a] Quantifier transitions.

[b] Qualifier transitions.

[c] The three decimal places were necessary in order to differentiate these transitions from those of the corresponding STDs.

Lipid species		Precursor ion type	Precursor ion *m/z* (Q1)	CEn (V)	Product ion *m/z* (Q3)	Product ion type	Ritention time (min)	Window (sec)	For TGs only: lost chain (C)
STD	TG(17:0/17:0/17:0)[a]	$[M+NH_4]^+$	866.8	30	579.5	Precursor$^+$ -RCOOH -NH_3	10.27	40	
STD	TG(17:0/17:0/17:0)[b]	$[M+NH_4]^+$	866.8	45	253.3	RCO^+	10.27	40	
STD	1,2-PC(17:0/17:0)[a]	$[M+H]^+$	762.6	42	184.1	$[Phosphocholine]^+$	5.46	90	
STD	1,2-PC(17:0/17:0)[b]	$[M+H]^+$	762.6	80	125.0	$[Phosphocholine –N(Me)_3]^+$	5.46	90	
STD	1,2-PE(15:0/15:0)[a]	$[M+H]^+$	664.5	25	523.5	Precursor$^+$ -phosphoethanolamine	4.07	60	
STD	1,2-PE(15:0/15:0)[b]	$[M+H]^+$	664.5	44	225.2	RCO^+	4.07	60	
STD	CE(17:0)[a]	$[M+NH_4]^+$	656.6	15	369.4	Precursor$^+$ -RCOOH -NH_3	9.82	40	
STD	SM(d18:1/16:0)[a]	$[M+H]^+$	703.6	36	184.1	$[Phosphocholine]^+$	4.24	90	
STD	SM(d18:1/16:0)[b]	$[M+H]^+$	703.6	36	125.0	$[Phosphocholine –N(Me)_3]^+$	4.24	90	

STD	Cer(d18:1/17:0)[a]	$[M+H]^+$	552.5	29	264.3	Precursor$^+$ -RCOOH -H_2O	4.96	40	
STD	Cer(d18:1/17:0)[b]	$[M+H]^+$	552.5	29	282.3	Precursor$^+$ -RCOOH	4.96	40	
STD	ISTD DMS(d18:1)[a]	$[M+H]^+$	328.3	26	310.3	Precursor$^+$ -H_2O	1.31	60	
STD	ISTD DMS(d18:1)[b]	$[M+H]^+$	328.3	26	280.3	Precursor$^+$ -CH_2O -H_2O	1.31	60	
1	TG(50:1) TG(16:0/16:0/18:1)	$[M+NH_4]^+$	850.8	30	577.5	Precursor$^+$ -RCOOH -NH_3	9.90	90	-C1 or C2
			850.8		551.5	Precursor$^+$ -RCOOH -NH_3			-C3
2	TG(50:2) TG(16:0/16:1/18:1)	$[M+NH_4]^+$	848.8	30	575.5	Precursor$^+$ -RCOOH -NH_3	9.67	60	-C1
			848.8		577.5	Precursor$^+$ -RCOOH -NH_3			-C2
			848.8		549.5	Precursor$^+$ -RCOOH -NH_3			-C3
3	TG(52:1) TG(18:1/18:0/16:0)	$[M+NH_4]^+$	878.8	30	579.5	Precursor$^+$ -RCOOH -NH_3	10.22	60	-C1
			878.8		577.5	Precursor$^+$ -RCOOH -NH_3			-C2
			878.8		605.6	Precursor$^+$ -RCOOH -NH_3			-C3
4	TG(52:3) TG(18:1/18:1/16:1)	$[M+NH_4]^+$	874.8	30	575.5	Precursor$^+$ -RCOOH -NH_3	9.77	60	-C1 or C2
			874.8		603.5	Precursor$^+$ -RCOOH -NH_3			-C3
5	TG(54:3) TG(18:1/18:1/18:1)	$[M+NH_4]^+$	902.8	30	603.5	Precursor$^+$ -RCOOH -NH_3	10.10	90	-C1 or C2 or C3
6	PC(32:0)	$[M+H]^+$	734.6	42	184.1	[Phosphocholine]$^+$	4.65	90	
7	PC(34:0)	$[M+H]^+$	762.601[c]	42	184.101[#]	[Phosphocholine]$^+$	4.88	90	
8	PC(34:3)	$[M+H]^+$	756.6	42	184.1	[Phosphocholine]$^+$	4.12	80	
9	PC(36:0)	$[M+H]^+$	790.6	42	184.1	[Phosphocholine]$^+$	5.54	60	
10	PC(36:1)	$[M+H]^+$	788.6	42	184.1	[Phosphocholine]$^+$	5.55	60	
11	PC(36:5)	$[M+H]^+$	780.6	42	184.1	[Phosphocholine]$^+$	4.11	90	

12	PC(38:2)	$[M+H]^+$	814.6	42	184.1	$[Phosphocholine]^+$	6.11	90	
13	PC(38:5)	$[M+H]^+$	808.6	42	184.1	$[Phosphocholine]^+$	4.68	90	
14	PC(38:6)	$[M+H]^+$	806.6	42	184.1	$[Phosphocholine]^+$	4.34	90	
15	PC(40:4)	$[M+H]^+$	838.6	42	184.1	$[Phosphocholine]^+$	5.60	90	
16	PC(40:5)	$[M+H]^+$	836.6	42	184.1	$[Phosphocholine]^+$	5.22	90	
17	PC(40:6)	$[M+H]^+$	834.6	42	184.1	$[Phosphocholine]^+$	5.00	90	
18	PC(40:7)	$[M+H]^+$	832.6	42	184.1	$[Phosphocholine]^+$	4.57	90	
19	CE(14:0)	$[M+NH_4]^+$	614.6	15	369.4	$Precursor^+$ -RCOOH -NH_3	9.17	40	
20	CE(16:0)	$[M+NH_4]^+$	642.6	15	369.4	$Precursor^+$ -RCOOH -NH_3	9.60	90	
21	CE(16:1)	$[M+NH_4]^+$	640.6	15	369.4	$Precursor^+$ -RCOOH -NH_3	9.27	60	
22	CE(18:0)	$[M+NH_4]^+$	670.6	15	369.4	$Precursor^+$ -RCOOH -NH_3	10.00	60	
23	CE(18:3)	$[M+NH_4]^+$	664.6	15	369.4	$Precursor^+$ -RCOOH -NH_3	9.11	60	
24	CE(20:3)	$[M+NH_4]^+$	692.6	15	369.4	$Precursor^+$ -RCOOH -NH_3	9.54	60	
25	CE(20:4)	$[M+NH_4]^+$	690.6	15	369.4	$Precursor^+$ -RCOOH -NH_3	9.30	60	
26	CE(22:6)	$[M+NH_4]^+$	714.6	15	369.4	$Precursor^+$ -RCOOH -NH_3	9.19	60	
27	SM(32:1)	$[M+H]^+$	675.5	36	184.1	$[Phosphocholine]^+$	3.70	60	
28	SM(32:2)	$[M+H]^+$	673.5	36	184.1	$[Phosphocholine]^+$	3.22	60	
29	SM(34:2)	$[M+H]^+$	701.6	36	184.1	$[Phosphocholine]^+$	3.76	90	
30	SM(35:1)	$[M+H]^+$	717.6	36	184.1	$[Phosphocholine]^+$	4.34	90	
31	SM(36:1)	$[M+H]^+$	731.6	36	184.1	$[Phosphocholine]^+$	4.72	40	
32	SM(36:2)	$[M+H]^+$	729.6	36	184.1	$[Phosphocholine]^+$	4.28	60	

33	SM(37:1)	$[M+H]^+$	745.6	36	184.1	$[Phosphocholine]^+$	4.96	90	
34	SM(38:1)	$[M+H]^+$	759.6	36	184.1	$[Phosphocholine]^+$	5.44	60	
35	SM(38:2)	$[M+H]^+$	757.6	36	184.1	$[Phosphocholine]^+$	4.42	60	
36	SM(38:3)	$[M+H]^+$	755.6	36	184.1	$[Phosphocholine]^+$	4.03	90	
37	SM(40:1)	$[M+H]^+$	787.7	36	184.1	$[Phosphocholine]^+$	6.04	90	
38	SM(40:2)	$[M+H]^+$	785.7	36	184.1	$[Phosphocholine]^+$	5.57	90	
39	SM(41:1)	$[M+H]^+$	801.7	36	184.1	$[Phosphocholine]^+$	6.48	90	
40	SM(41:2)	$[M+H]^+$	799.7	36	184.1	$[Phosphocholine]^+$	5.94	90	
41	SM(42:1)	$[M+H]^+$	815.7	36	184.1	$[Phosphocholine]^+$	6.79	90	
42	SM(42:3)	$[M+H]^+$	811.7	36	184.1	$[Phosphocholine]^+$	5.68	90	
43	SM(42:4)	$[M+H]^+$	809.7	36	184.1	$[Phosphocholine]^+$	5.09	90	
44	SM(43:1)	$[M+H]^+$	829.7	36	184.1	$[Phosphocholine]^+$	7.13	90	
45	SM(43:2)	$[M+H]^+$	827.7	36	184.1	$[Phosphocholine]^+$	6.47	90	
46	SM(43:3)	$[M+H]^+$	825.7	36	184.1	$[Phosphocholine]^+$	6.03	90	
47	PE(34:0)	$[M+H]^+$	720.6	25	579.5	$Precursor^+$ -phosphoethanolamine	4.82	90	
48	PE(34:1)	$[M+H]^+$	718.5	25	577.5	$Precursor^+$ -phosphoethanolamine	4.83	90	
49	PE(34:2)	$[M+H]^+$	716.5	25	575.5	$Precursor^+$ -phosphoethanolamine	4.45	60	
50	PE(36:0)	$[M+H]^+$	748.6	25	607.6	$Precursor^+$ -phosphoethanolamine	5.20	90	
51	PE(36:1)	$[M+H]^+$	746.6	25	605.6	$Precursor^+$ -phosphoethanolamine	5.44	90	
52	PE(36:2)	$[M+H]^+$	744.6	25	603.5	$Precursor^+$ -phosphoethanolamine	5.05	90	
53	PE(36:3)	$[M+H]^+$	742.5	25	601.5	$Precursor^+$ -phosphoethanolamine	4.64	60	

54	PE(36:4)	$[M+H]^+$	740.5	25	599.5	Precursor$^+$ -phosphoethanolamine	4.47	60	
55	PE(36:5)	$[M+H]^+$	738.5	25	597.5	Precursor$^+$ -phosphoethanolamine	4.10	90	
56	PE(38:1)	$[M+H]^+$	774.6	25	633.6	Precursor$^+$ -phosphoethanolamine	6.53	90	
57	PE(38:2)	$[M+H]^+$	772.6	25	631.6	Precursor$^+$ -phosphoethanolamine	6.06	90	
58	PE(38:3)	$[M+H]^+$	770.6	25	629.6	Precursor$^+$ -phosphoethanolamine	5.28	90	
59	PE(38:5)	$[M+H]^+$	766.5	25	625.5	Precursor$^+$ -phosphoethanolamine	4.66	90	
60	PE(38:6)	$[M+H]^+$	764.5	25	623.5	Precursor$^+$ -phosphoethanolamine	4.34	60	
61	PE(40:4)	$[M+H]^+$	796.6	25	655.6	Precursor$^+$ -phosphoethanolamine	5.54	90	
62	PE(40:5)	$[M+H]^+$	794.6	25	653.6	Precursor$^+$ -phosphoethanolamine	4.98	90	
63	PE(40:6)	$[M+H]^+$	792.6	25	651.5	Precursor$^+$ -phosphoethanolamine	4.97	90	
64	PE(40:7)	$[M+H]^+$	790.5	25	649.5	Precursor$^+$ -phosphoethanolamine	4.57	90	
65	Cer(d18:0/24:0)	$[M+H]^+$	652.7	29	266.3	Precursor$^+$ -RCOOH -H_2O	7.08	90	
66	Cer(d18:1/16:0)	$[M+H]^+$	538.5	29	264.3	Precursor$^+$ -RCOOH -H_2O	4.62	40	
67	Cer(d18:1/18:0)	$[M+H]^+$	566.6	29	264.3	Precursor$^+$ -RCOOH -H_2O	5.25	50	
68	Cer(d18:1/22:0)	$[M+H]^+$	622.6	29	264.3	Precursor$^+$ -RCOOH -H_2O	6.50	40	
69	Cer(d18:1/24:0)	$[M+H]^+$	650.6	29	264.3	Precursor$^+$ -RCOOH -H_2O	7.07	40	

Table S2. Equations and R^2 obtained for the calibration curves of lipid classes.

$y = 0.1152x – 0.0158$ with $R^2 = 0.9964$	Cer(d18:1/17:0)
$y = 0.0991x – 0.0454$ with $R^2 = 0.9948$	1,2-PE(15:0/15:0)
$y = 0.5444x – 0.2006$ with $R^2 = 0.9969$	1,2-PC(17:0/17:0)
$y = 0.3287x – 0.1639$ with $R^2 = 0.9990$	TG(17:0/17:0/17:0)
$y = 0.1608x + 13.866$ with $R^2 = 0.9485$	CE(17:0)
$y = 0.3137x – 0.3524$ with $R^2 = 0.9973$	SM(d18:1/16:0)

Table S3. CTA score: classification and patient characteristics.

Continuous variables are reported as means with relative standard deviation and p-values are referred to one-way ANOVA test. For continuous variable resulted significantly different (ANOVA p-value < 0.05) a two-tailed Student T-test with Bonferroni correction was also performed in order to compare each group of subjects with respect to the control group (Class No CAD). Test T p-value is shown only where significance emerged. Categorical variables are reported as numbers and relative percentages and p-values are referred to Chi-square (χ^2) test.

	**No CAD** CTA score = 0 N = 10	**Class 1** CTA score < 5 N = 12	**Class 2** CTA score = 5-20 N = 67	**Class 3** CTA Score > 20 N = 43	**ANOVA or χ^2 p-value**	**test T p-value**
**Age** (yrs)	**65** ± 7.6	**66** ± 5.3	**67.6** ± 8.2	**72.3** ± 6.2	** 0.002	2 vs No: * 0.031
**Male sex**	2 (**20%**)	7 (**58.3%**)	40 (**59.7%**)	36 (**83.7%**)	** 0.001	
**Family History of CHD**	6 (**60%**)	4 (**33.3%**)	30 (**44.8%**)	21 (**48.8%**)	0.628	
**Smoke**	1 (**10%**)	0 (**0%**)	11 (**16.4%**)	3 (**7%**)	0.253	
**Diabete Mellitus**	2 (**20%**)	1 (**8.3%**)	18 (**26.9%**)	19 (**44.2%**)	0.056	
**Dyslipidemia**	10 (**100%**)	11 (**91.7%**)	64 (**95.5%**)	36 (**83.7%**)	0.124	
**Hypertension**	8 (**80%**)	10 (**83.3%**)	53 (**79.1%**)	34 (**79.1%**)	0.989	
**Obesity**	3 (**30%**)	3 (**25%**)	14 (**20.9%**)	10 (**23.3%**)	0.925	
**Daily Physical Activity**	4 (**40%**)	7 (**58.3%**)	49 (**73.1%**)	30 (**69.8%**)	0.145	
**Symptoms**	7 (**70%**)	5 (**41.7%**)	26 (**38.8%**)	14 (**32.6%**)	0.187	
**Total Cholesterol** (mg/dL)	**171.4** ± 18.6	**175.7** ± 50.6	**173.3** ± 34.2	**157.9** ± 31.5	0.116	
**LDL-C** (mg/dL)	**85.8** ± 18.3	**94** ± 40.4	**89.8** ± 29.5	**77.1** ± 33.2	0.159	
**HDL-C** (mg/dL)	**62.6** ± 15.7	**58.7** ± 19.1	**56** ± 14.7	**52.1** ± 10.7	0.127	
**nonHDL-C** (mg/dL)	**108.8** ± 20.1	**117** ± 40.3	**117.3** ± 29.7	**105.8** ± 30.8	0.254	
**Triglycerides** (mg/dL)	**115.3** ± 50.3	**119.1** ± 43.7	**142.4** ± 65.2	**166.3** ± 139.7	0.256	

Clinical variables are defined as follow: Smoke, current smoking; Diabete Mellitus, Fasting glucose > 126 mg/dl and/or GHbA1c > 6.5% or under treatment; Dyslipidemia, LDL > 120 mg/dl, HDL < 40 for man or < 50 for woman, Triglycerides > 150 mg/dL or under specific treatments; Hypertension, Systolic and diastolic pressure >140/90 mmHg or under treatment; Obesity, BMI Kg/m^2 > 30 and /or waist circ > 102 cm for man or > 88 cm for woman; Daily Physical Activity, at least one hour of gentle walking per day; Symptoms, include typical angina, atypical angina, non angina chest pain.

CHD, coronary heart disease; LDL-C, low-density lipoprotein cholesterol; HDL-C, high density lipoprotein cholesterol.

Table S4. LPP (lipid plaque prevalence) ratio (non-calcified + mixed plaques /total number of plaques): classification and patient characteristics.
For variable definitions and relative p-values see Table S3 heading and text below.

	**No CAD** No Plaques N = 10	**Class 0** LPP = 0 N = 21	**Class 1** LPP = 0.10-0.40 N = 33	**Class 2** LPP = 0.41-0.63 N = 34	**Class 3** LPP = 0.64-1 N = 34	**ANOVA or χ^2 p-value**	**test T p-value**
**Age** (yrs)	**65** ± 7.6	**65.6** ± 7	**69.2** ± 7.6	**71.9** ± 7	**68.4** ± 7.8	*0.014	n.s. vs No
**Male sex**	2 (**20%**)	14 (**66.7%**)	21 (**63.6%**)	27 (**79.4%**)	21 (**61.8%**)	*0.017	
**Family History of CHD**	6 (**60%**)	10 (**47.6%**)	13 (**39.4%**)	14 (**41.2%**)	18 (**52.9%**)	0.669	
**Smoke**	1 (**10%**)	4 (**19%**)	2 (**6.1%**)	5 (**14.7%**)	3 (**8.8%**)	0.598	
**Diabete Mellitus**	2 (**20%**)	2 (**9.5%**)	13 (**39.4%**)	10 (**29.4%**)	13 (**38.2%**)	0.130	
**Dyslipidemia**	10 (**100%**)	19 (**90.5%**)	32 (**97%**)	28 (**82.4%**)	32 (**94.1%**)	0.178	
**Hypertension**	8 (**80%**)	14 (**66.7%**)	28 (**84.8%**)	30 (**88.2%**)	25 (**73.5%**)	0.283	
**Obesity**	3 (**30%**)	4 (**19%**)	9 (**27.3%**)	9 (**26.5%**)	5 (**14.7%**)	0.668	
**Daily Physical Activity**	4 (**40%**)	14 (**66.7%**)	24 (**72.7%**)	24 (**70.6%**)	24 (**70.6%**)	0.351	
**Symptoms**	7 (**70%**)	10 (**47.6%**)	10 (**30.3%**)	10 (**29.4%**)	15 (**44.1%**)	0.116	
**Total Cholesterol** (mg/dL)	**171.4** ± 18.6	**172.1** ± 36.3	**164.9** ± 31.1	**160.6** ± 35.3	**176.2** ± 39.3	0.401	
**LDL-C** (mg/dL)	**85.8** ± 18.3	**88.6** ± 27.1	**78** ± 24.1	**81.2** ± 37.1	**95.9** ± 35.6	0.169	
**HDL-C** (mg/dL)	**62.6** ± 15.7	**58.5** ± 15.3	**57.6** ± 16.6	**53** ± 12.2	**51.9** ± 11.3	0.111	
**nonHDL-C** (mg/dL)	**108.8** ± 20.1	**113.6** ± 29.9	**107.4** ± 23.3	**107.6** ± 33.3	**124.3** ± 35.4	0.136	
**Triglycerides** (mg/dL)	**115.3** ± 50.3	**124.5** ± 78.6	**147** ± 57.5	**160.4** ± 153.1	**153.1** ± 62	0.548	

Table S5. CAD-RADS: classification and patient characteristics.

For variable definitions and relative p-values see Table S3 heading and text below.

	**No CAD** (no stenosis) N = 10	**Class 1** (< 30% stenosis) N = 39	**Class 2** (30%-50% stenosis) N = 29	**Class 3** (> 50% stenosis) N = 54	**ANOVA or χ^2 p-value**	**test T p-value**
**Age** (yrs)	**65** ± 7.6	**64.7** ± 7.2	**70** ± 8.2	**71.9** ± 6.2	*** < 0.001	3 vs No: * 0.033
**Male sex**	2 (**20%**)	27 (**69.2%**)	13 (**44.8%**)	43 (**79.6%**)	*** < 0.001	
**Family History of CHD**	6 (**60%**)	18 (**46.2%**)	10 (**34.5%**)	27 (**50%**)	0.443	
**Smoke**	1 (**10%**)	4 (**10.3%**)	4 (**13.8%**)	6 (**11.1%**)	0.971	
**Diabete Mellitus**	2 (**20%**)	5 (**12.8%**)	11 (**37.9%**)	22 (**40.7%**)	* 0.021	
**Dyslipidemia**	10 (**100%**)	37 (**94.9%**)	28 (**96.6%**)	46 (**85.2%**)	0.151	
**Hypertension**	8 (**80%**)	28 (**71.8%**)	26 (**89.7%**)	43 (**79.6%**)	0.353	
**Obesity**	3 (**30%**)	8 (**20.5%**)	8 (**27.6%**)	11 (**20.4%**)	0.808	
**Daily Physical Activity**	4 (**40%**)	29 (**74.4%**)	19 (**65.5%**)	38 (**70.4%**)	0.189	
**Symptoms**	7 (**70%**)	16 (**41%**)	12 (**41.4%**)	17 (**31.5%**)	0.143	
**Total Cholesterol** (mg/dL)	**171.4** ± 18.6	**171.8** ± 32	**182.4** ± 45.4	**157.7** ± 29.3	* 0.015	n.s. vs No
**LDL-C** (mg/dL)	**85.8** ± 18.3	**88.3** ± 26.8	**98.3** ± 43.6	**77.1** ± 26.6	* 0.028	n.s. vs No
**HDL-C** (mg/dL)	**62.6** ± 15.7	**57** ± 14.7	**55.2** ± 17	**53.1** ± 11.4	0.219	
**nonHDL-C** (mg/dL)	**108.8** ± 20.1	**114.8** ± 28.2	**127.2** ± 38.2	**104.6** ± 27	0.013	
**Triglycerides** (mg/dL)	**115.3** ± 50.3	**133.8** ± 61.2	**177.2** ± 161.1	**143.8** ± 68	0.184	

Table S6. Means and standard deviations (SD) (μM) for 69 quantified lipid species among groups of patients defined by CTA score annotations.
The degrees of variation evaluated as log_2 trasformed fold change (FC = Class mean /No CAD mean) together with their significance after correction using the Benjamini-Hochberg procedure are shown.

	No CAD		Class 1				Class 2				Class 3			
	**Mean**	**SD**	**Mean**	**SD**	**logFC**	**adj p-val**	**Mean**	**SD**	**logFC**	**adj p-val**	**Mean**	**SD**	**logFC**	**adj p-val**
**Cer(d18:0/24:0)**	0.12	0.05	0.14	0.05	0.22	0.797	0.14	0.06	0.22	0.472	0.15	0.07	0.24	0.447
**Cer(d18:1/16:0)**	0.92	1.16	0.70	1.00	-0.39	0.768	0.53	0.57	-0.80	0.470	0.54	0.35	-0.77	0.720
**Cer(d18:1/18:0)**	0.11	0.04	0.13	0.08	0.23	0.974	0.10	0.05	-0.21	0.283	0.10	0.04	-0.17	0.315
**Cer(d18:1/22:0)**	0.21	0.06	0.22	0.07	0.05	0.974	0.24	0.08	0.19	0.484	0.23	0.08	0.12	0.679
**Cer(d18:1/24:0)**	0.70	0.22	0.67	0.27	-0.06	0.974	0.74	0.26	0.07	0.798	0.68	0.25	-0.05	0.677
**CE(14:0)**	26.43	8.54	27.72	9.57	0.07	0.974	32.95	10.73	0.32	0.206	31.82	13.25	0.27	0.315
**CE(16:0)**	621.96	211.71	407.79	184.68	-0.61	0.408	474.88	239.48	-0.39	0.207	423.45	249.64	-0.55	0.034
**CE(16:1)**	82.79	16.16	90.08	17.49	0.12	0.721	91.96	24.21	0.15	0.360	103.59	70.37	0.32	0.260
**CE(18:0)**	28.73	8.41	23.24	6.97	-0.31	0.493	24.63	8.10	-0.22	0.277	21.22	6.32	-0.44	0.030
**CE(18:3)**	89.60	14.23	82.54	23.52	-0.12	0.866	93.93	19.41	0.07	0.557	91.21	23.83	0.03	0.678
**CE(20:3)**	34.57	9.81	35.12	6.41	0.02	0.826	37.92	10.03	0.13	0.471	36.42	9.74	0.08	0.520
**CE(20:4)**	562.00	266.72	304.36	244.99	-0.88	0.408	362.03	284.14	-0.63	0.084	317.93	290.06	-0.82	0.024
**CE(22:6)**	42.26	11.78	34.95	10.70	-0.27	0.690	34.96	12.85	-0.27	0.206	36.06	12.82	-0.23	0.280
**PE(34:0)**	4.69	0.24	4.81	0.29	0.04	0.878	4.99	0.47	0.09	0.206	5.12	0.54	0.13	0.037

**PE(34:1)**	8.59	1.60	9.54	1.86	0.15	0.768	11.09	3.44	0.37	0.084	12.31	4.11	0.52	0.009
**PE(34:2)**	9.44	2.25	10.02	2.00	0.09	0.837	12.28	4.60	0.38	0.207	13.70	6.61	0.54	0.030
**PE(36:0)**	6.79	0.36	6.88	1.42	0.02	0.974	6.86	0.96	0.02	1.000	7.13	1.52	0.07	0.679
**PE(36:1)**	12.30	2.57	14.84	4.25	0.27	0.837	17.78	6.85	0.53	0.084	19.95	9.09	0.70	0.019
**PE(36:2)**	25.39	8.23	29.43	8.66	0.21	0.768	36.09	16.04	0.51	0.205	41.62	20.67	0.71	0.024
**PE(36:3)**	10.54	2.33	11.37	2.53	0.11	0.797	13.95	5.36	0.40	0.227	15.93	6.33	0.60	0.030
**PE(36:4)**	15.74	4.46	15.10	3.50	-0.06	0.923	18.84	7.10	0.26	0.485	20.39	5.95	0.37	0.064
**PE(36:5)**	4.91	0.57	5.02	0.66	0.03	0.974	5.44	1.17	0.15	0.283	5.78	1.35	0.23	0.085
**PE(38:1)**	5.20	0.30	5.09	0.51	-0.03	0.721	5.09	0.43	-0.03	0.426	5.09	0.34	-0.03	0.434
**PE(38:2)**	6.51	0.65	6.51	0.70	0.00	0.974	6.89	1.00	0.08	0.448	7.14	1.16	0.13	0.129
**PE(38:3)**	17.60	4.76	16.59	3.72	-0.08	0.837	19.37	6.24	0.14	0.715	20.42	7.17	0.21	0.434
**PE(38:5)**	19.32	5.79	18.30	4.16	-0.08	0.878	21.88	8.49	0.18	0.557	23.69	7.28	0.29	0.176
**PE(38:6)**	28.31	14.06	23.69	9.10	-0.26	0.768	28.76	12.56	0.02	0.816	32.41	14.59	0.19	0.447
**PE(40:4)**	6.73	1.44	6.95	1.61	0.05	0.974	7.49	1.66	0.15	0.277	8.00	2.24	0.25	0.078
**PE(40:5)**	10.70	2.45	10.80	2.48	0.01	0.974	12.04	3.54	0.17	0.486	12.77	4.08	0.25	0.259
**PE(40:6)**	24.17	12.35	21.76	8.96	-0.15	0.878	24.21	9.36	0.00	0.753	27.02	11.87	0.16	0.447
**PE(40:7)**	7.23	1.72	6.61	1.20	-0.13	0.768	7.26	1.57	0.01	0.957	7.98	1.77	0.14	0.315
**PC(32:0)**	34.81	5.53	35.67	6.84	0.04	0.974	35.36	8.06	0.02	1.000	36.42	7.80	0.07	0.499
**PC(34:0)**	50.48	7.14	54.94	12.01	0.12	0.697	56.33	12.50	0.16	0.277	57.77	12.93	0.19	0.123
**PC(34:3)**	41.43	9.99	38.37	11.91	-0.11	0.826	43.96	15.92	0.09	0.919	42.78	15.79	0.05	1.000
**PC(36:0)**	13.20	2.29	15.65	5.77	0.24	0.697	16.83	5.76	0.35	0.206	17.24	6.30	0.38	0.085

**PC(36:1)**	120.51	23.19	135.75	25.62	0.17	0.690	159.57	75.25	0.41	0.206	161.40	75.69	0.42	0.153
**PC(36:5)**	74.47	24.12	71.41	33.88	-0.06	0.974	77.35	34.99	0.05	1.000	81.30	37.70	0.13	0.782
**PC(38:2)**	69.30	12.34	65.62	15.62	-0.08	0.974	58.48	13.37	-0.24	0.084	57.08	10.11	-0.28	0.030
**PC(38:5)**	229.32	106.35	169.45	65.31	-0.44	0.768	181.17	83.07	-0.34	0.292	183.23	83.81	-0.32	0.251
**PC(38:6)**	189.30	97.37	144.98	59.55	-0.38	0.768	151.23	58.02	-0.32	0.442	162.51	74.24	-0.22	0.514
**PC(40:4)**	17.71	4.55	15.31	7.51	-0.21	0.408	17.34	5.68	-0.03	0.702	15.64	5.15	-0.18	0.280
**PC(40:5)**	47.07	10.20	41.79	12.22	-0.17	0.605	43.95	11.45	-0.10	0.544	42.13	11.28	-0.16	0.280
**PC(40:6)**	90.36	19.03	75.93	20.39	-0.25	0.697	79.90	19.68	-0.18	0.318	79.31	20.14	-0.19	0.204
**PC(40:7)**	17.47	4.54	13.42	3.92	-0.38	0.408	14.37	4.76	-0.28	0.206	15.59	4.62	-0.16	0.339
**TG(50:1)**	162.20	63.11	181.03	69.55	0.16	0.866	217.24	80.03	0.42	0.206	221.61	78.22	0.45	0.074
**TG(50:2)**	85.29	37.64	79.86	30.89	-0.09	0.974	103.19	44.66	0.27	0.341	103.75	46.49	0.28	0.315
**TG(52:1)**	94.55	34.29	116.01	41.60	0.30	0.690	135.51	62.21	0.52	0.166	152.62	72.85	0.69	0.024
**TG(52:3)**	122.30	42.23	105.45	24.93	-0.21	0.768	113.46	25.80	-0.11	0.965	115.68	21.24	-0.08	0.766
**TG(54:3)**	90.58	29.60	92.66	21.62	0.03	0.974	105.12	67.25	0.21	0.779	111.19	49.54	0.30	0.285
**SM(32:2)**	1.58	0.31	1.33	0.32	-0.25	0.647	1.37	0.44	-0.21	0.206	1.27	0.50	-0.32	0.034
**SM(32:1)**	10.81	2.57	9.80	1.97	-0.14	0.768	11.02	3.26	0.03	1.000	11.13	3.36	0.04	0.809
**SM(34:2)**	30.19	5.06	24.72	4.53	-0.29	0.408	24.02	4.84	-0.33	0.044	23.18	2.96	-0.38	0.003
**SM(35:1)**	6.67	1.57	5.54	1.39	-0.27	0.493	5.82	1.53	-0.20	0.279	5.24	1.38	-0.35	0.034
**SM(36:2)**	26.64	7.40	22.40	6.52	-0.25	0.697	19.65	5.30	-0.44	0.044	18.19	4.35	-0.55	0.004
**SM(36:1)**	45.90	7.96	42.72	9.35	-0.10	0.974	40.05	8.45	-0.20	0.206	37.81	7.42	-0.28	0.041
**SM(37:1)**	15.83	5.05	13.36	3.00	-0.25	0.697	13.59	4.11	-0.22	0.279	12.57	2.75	-0.33	0.085

**SM(38:3)**	4.12	0.98	3.52	1.25	-0.23	0.690	4.21	1.47	0.03	0.965	3.50	1.22	-0.24	0.249
**SM(38:2)**	16.60	4.69	13.50	3.41	-0.30	0.493	12.23	3.22	-0.44	0.044	11.04	2.18	-0.59	0.003
**SM(38:1)**	51.23	8.65	45.43	9.60	-0.17	0.697	47.78	10.22	-0.10	0.405	43.75	7.97	-0.23	0.045
**SM(40:2)**	77.44	14.71	63.08	13.58	-0.30	0.408	63.79	13.01	-0.28	0.084	58.14	10.24	-0.41	0.003
**SM(40:1)**	121.70	18.59	105.04	23.53	-0.21	0.493	109.75	31.92	-0.15	0.277	102.24	19.42	-0.25	0.030
**SM(41:2)**	55.35	13.76	42.53	12.05	-0.38	0.408	44.64	12.36	-0.31	0.171	39.80	10.22	-0.48	0.017
**SM(41:1)**	62.95	17.25	49.59	16.62	-0.34	0.408	55.08	14.61	-0.19	0.350	46.61	12.11	-0.43	0.024
**SM(42:4)**	12.26	2.55	9.50	2.33	-0.37	0.408	9.48	2.61	-0.37	0.044	8.60	1.94	-0.51	0.003
**SM(42:3)**	122.77	20.59	103.12	21.51	-0.25	0.408	97.31	22.83	-0.34	0.044	91.51	14.57	-0.42	0.003
**SM(42:1)**	89.07	20.21	75.94	21.74	-0.23	0.697	80.06	19.20	-0.15	0.336	71.01	18.37	-0.33	0.030
**SM(43:3)**	6.52	1.49	5.25	1.39	-0.31	0.434	5.63	1.79	-0.21	0.207	5.13	1.35	-0.35	0.033
**SM(43:2)**	12.90	3.74	11.34	4.24	-0.19	0.768	10.96	4.17	-0.24	0.279	10.06	3.77	-0.36	0.078
**SM(43:1)**	4,73	2,15	4,32	1,43	-0.13	0.768	4.21	1.78	-0.17	0.509	3.55	1.26	-0.41	0.122

Figure S1. Lipid profile analysis based on LPP ratio annotation.

Volcano plots showing the differentially expressed lipids (adj. p-value threshold = 0.05) among LPP ratio annotation Classes relative to No CAD subjects. Lipid species are colored according to lipid class (CE, Cer, PC, PE, SM, TG). The panels on the left report in details lipids species resulted significantly dysregulated in comparison Class 1 vs No CAD, Class 2 vs No CAD and Class 3 vs No CAD.

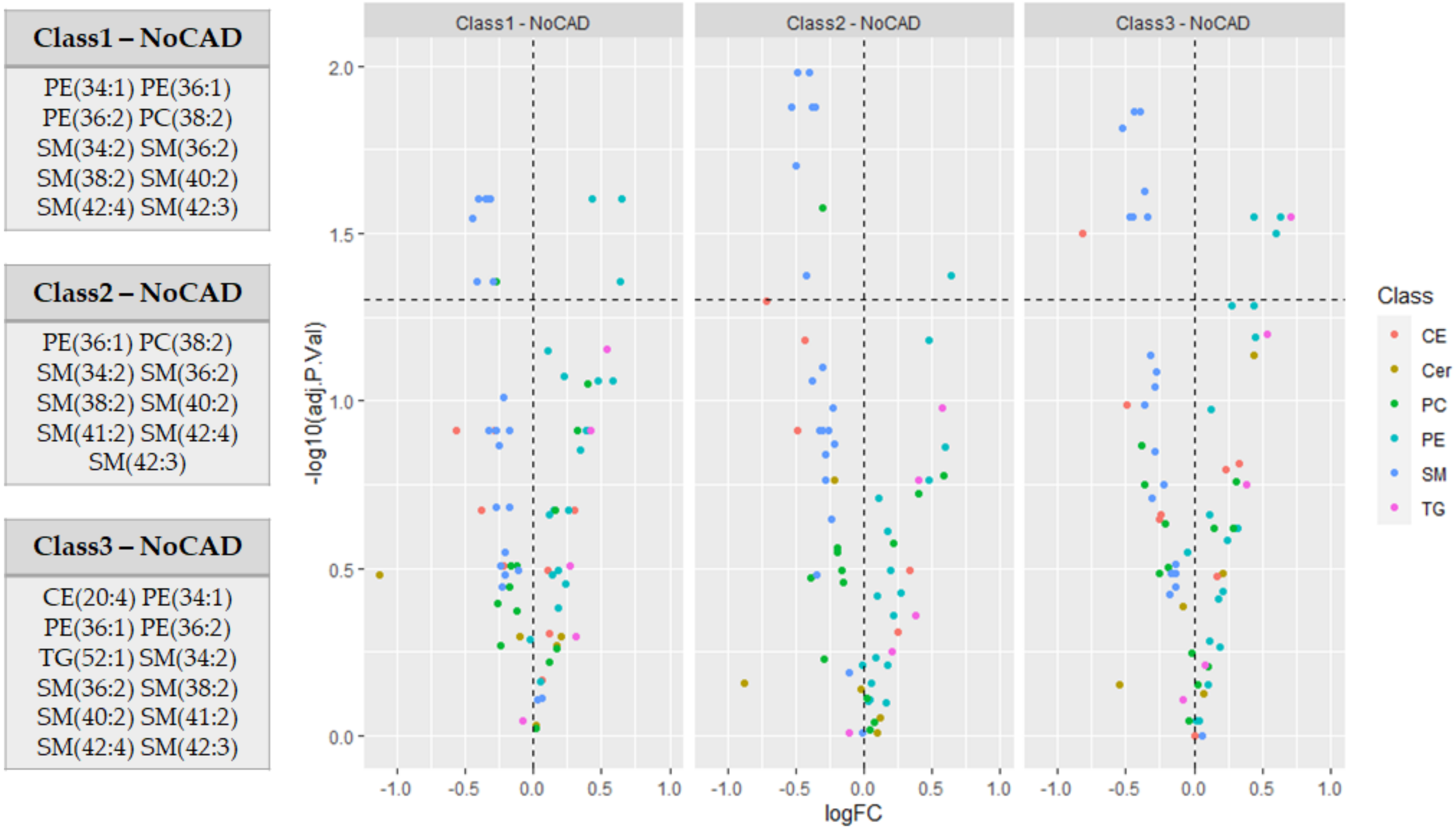

Figure S2. Lipid profile analysis based on CAD-RADS annotation.

Volcano plots showing the differentially expressed lipids (adj. p-value threshold = 0.05) among CAD-RADS annotation Classes relative to No CAD subjects. Lipid species are colored according to lipid class (CE, Cer, PC, PE, SM, TG). The panels on the left report in details lipids species which resulted significantly dysregulated in comparison Class 2 vs No CAD and Class 3 vs No CAD.

Class2 – NoCAD
CE(20:4)
PE(34:1)
PE(36:1)
PE(36:2)
TG(52:1)
SM(34:2)
SM(40:2)
SM(42:4)
SM(42:3)

Class3 – NoCAD
SM(34:2)
SM(36:2)
SM(38:2)
SM(40:2)
SM(41:2)
SM(42:4)
SM(42:3)

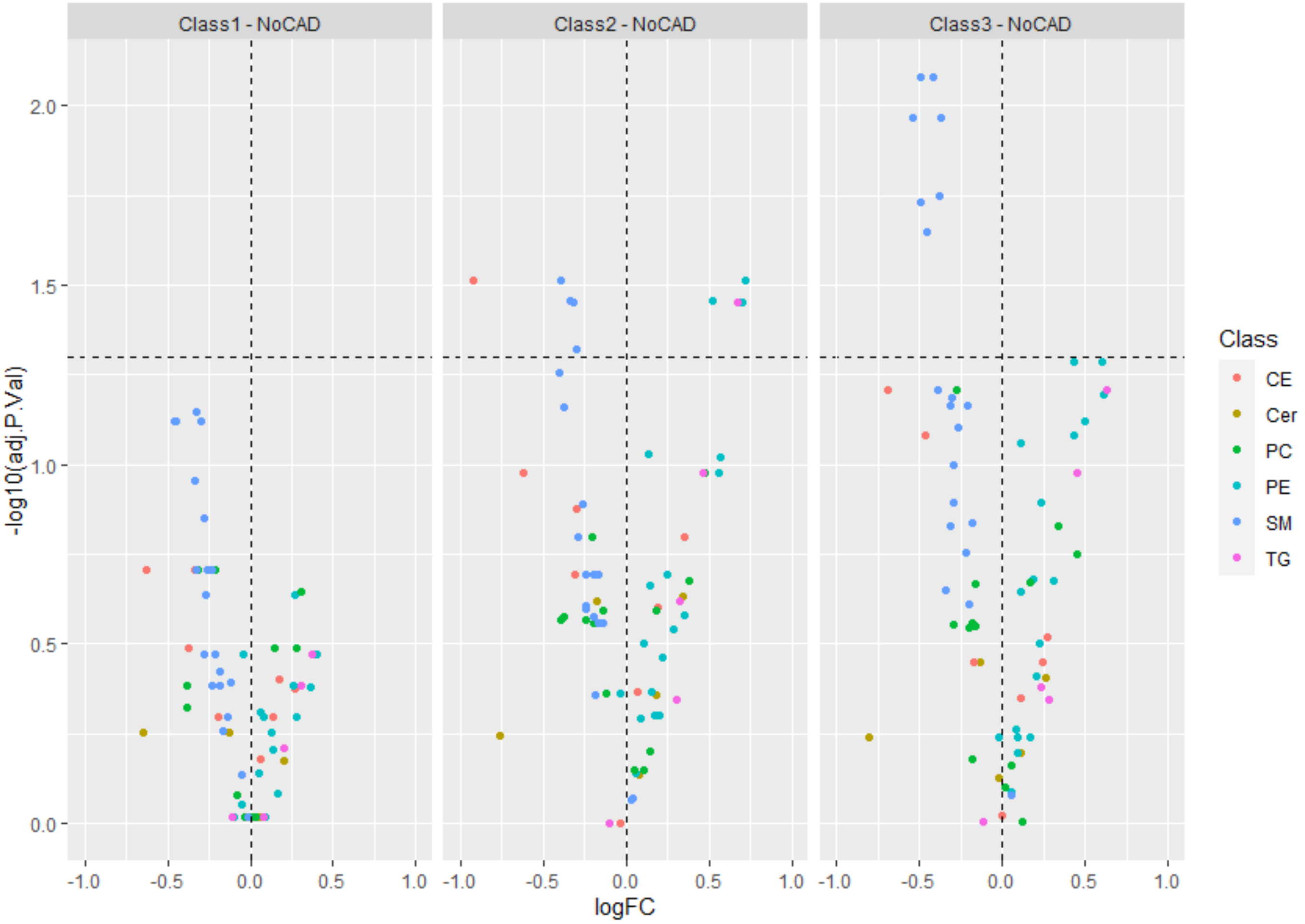

Supplement: Supplementary file 1 — Supplementary Information. [file 41598_2021_92339_MOESM1_ESM.pdf]
